# Identification, Subcellular Localization, and Infection-Related Expression of a Novel Haloacid Dehalogenase Gene (*VmHAD*) from *Valsa mali* Vm1

**DOI:** 10.3390/jof11120827

**Published:** 2025-11-23

**Authors:** Shuwu Zhang, Xingxu Chen, Cizhong Duan, Jia Liu, Fei Tao, Bingliang Xu

**Affiliations:** 1 Biocontrol Engineering Laboratory of Crop Diseases and Pests of Gansu Province, College of Plant Protection, Gansu Agricultural University, Lanzhou 730070, China; zhangsw704@126.com (S.Z.);; 2 State Key Laboratory of Aridland Crop Science, Gansu Agricultural University, Lanzhou 730070, China

**Keywords:** apple Valsa canker, bioinformation analysis, expression characteristics determination, gene cloning, haloacid dehalogenase enzyme, *Valsa mali*

## Abstract

The haloacid dehalogenase (HAD) superfamily represents a large group of enzymes across diverse taxa. However, the characteristics and functional roles of HAD members in the destructive apple canker pathogen, *Valsa mali* strain Vm1 (Vm1), remain poorly understood, particularly regarding their expression during infection. In this study, the full-length cDNA sequence of the *VmHAD* gene from Vm1 was cloned using rapid amplification of cDNA ends (RACE) technology, and its bioinformatic properties, subcellular localization, and expression patterns during infection were characterized. The *VmHAD* cDNA was 1044 bp in length, containing a complete open reading frame (ORF) of 798 bp that encodes a 265 amino acid protein with a conserved HAD-like domain. Phylogenetic analysis revealed that VmHAD shares the highest similarity with the (S)-2-haloacid dehalogenase (accession no. KUI70710.1) from *Cytospora mali* 03-8, belonging to the L-2-haloacid dehalogenase family within the HAD hydrolase superfamily. Subcellular localization analysis using a transient expression system in *Nicotiana benthamiana* indicated that VmHAD is distributed in both the nucleus and cytoplasm. Expression profiling demonstrated that *VmHAD* was significantly upregulated during the infection of detached apple branches by Vm1, with relative expression levels increasing 3.13-, 4.25-, and 3.98-fold at 3, 5, and 7 days post-inoculation, respectively, compared with day 1, whereas no expression was detected in the uninoculated control. These findings identify *VmHAD* as a novel HAD family member in Vm1 and suggest that it plays a potential role in the infection process and pathogenicity. This work provides new insights into the molecular mechanisms underlying *V. mali* pathogenicity and contributes to the development of effective strategies for disease management.

## 1. Introduction

Apples (*Malus domestica* Borkh.) are one of the most widely cultivated and economically important fruit crops worldwide [1,2,3]. Apple Valsa canker, caused by the fungal pathogen *Valsa mali*, is a destructive disease that severely reduces apple yield and quality, leading to significant economic losses in apple-producing regions [4,5,6]. Previous studies have reported disease incidences ranging from 50% to 100%, with complete yield losses in older orchards in East Asia [7,8,9,10,11,12,13]. Therefore, developing effective and sustainable management strategies against apple Valsa canker is of great importance.

Over the past decade, extensive research has focused on identifying the pathogenic factors and elucidating the infection mechanisms of *V. mali*. The virulence of *V. mali* has been attributed mainly to multiple pathogenic determinants, including cell wall-degrading enzymes (CWDEs), toxins, microRNA-like RNAs, effector proteins, secondary metabolites, and various pathogenic signaling regulators [14,15,16,17]. These factors collectively disrupt host cellular structure and metabolism, alter the chemical composition of apple tissues, and ultimately cause cell disintegration and branch decay. In particular, the secretion of CWDEs (such as xylanase, pectinase, and β-glucosidase) and the production of toxins (such as protocatechuic acid and *p*-hydroxybenzoic acid) are key virulence mechanisms that facilitate host cell wall degradation and tissue necrosis [15,18,19,20,21]. Several genes associated with these processes, including *VmXyl1*, *VmGlu2*, *VmXyl2*, *VmHbh1*, and *VmHbh4*, have been identified and characterized [22,23,24,25]. In our previous transcriptomic analysis of the *V. mali* strain Vm1 (Vm1), a gene encoding a haloacid dehalogenase (HAD) superfamily enzyme, designated *VmHAD*, was identified and annotated to several metabolic pathways. These findings suggested that *VmHAD* may play a role in virulence metabolism or stress response signaling during host infection. However, its biological characteristics and potential function in Vm1 pathogenesis remain unknown.

The HAD superfamily represents one of the largest and most diverse groups of hydrolases, comprising enzymes such as dehalogenases, ATPases, phosphatases, phosphomutases, and phosphonatases [26,27,28,29]. Members of this family are widely distributed among prokaryotic and eukaryotic organisms and participate in numerous metabolic and regulatory processes. Various HAD enzymes have been structurally and biochemically characterized from different organisms, including phosphoserine phosphatase (SerB), phosphoglycolate phosphatase, phosphonacetaldehyde hydrolase, phosphoglucomutase, and inorganic pyrophosphatase from *Methanococcus jannaschii* [30], *Thermoplasma acidophilum* [31], *Bacillus cereus* [32], *Lactococcus lactis* [33], and *Bacteroides thetaiotaomicron* BT2127 [34], as well as phosphatases YbiV and NagD from *Escherichia coli* K-12 [35,36]. Additionally, haloacid dehalogenases have been reported in *Pseudomonas* spp. [37,38], *Xanthobacter autotrophicus* [39], and *Staphylococcus lugdunensis* (SLHAD1) [40]. While these studies have mainly focused on structural and biochemical features and their applications in environmental bioremediation, little is known about HAD enzymes in phytopathogenic fungi. Only a few HAD-related genes have been reported in plant pathogens, including *cutA* from *Fusarium fujikuroi* [41], *FoHAD-type II* from *F. oxysporum* f. sp. *momordicae* [42], *FOXG-07877* from *F. oxysporum* f. sp. *fragariae* [43], and *Nem1* from *Botryosphaeria dothidea* [44].

However, the biological characteristics and specific functions of the *VmHAD* gene in Vm1, particularly its expression dynamics during infection, have not been elucidated. Therefore, the objectives of this study were to (i) clone the full-length cDNA sequence of *VmHAD* gene from Vm1 and analyze its bioinformatic features; (ii) construct a recombinant expression vector to assess subcellular localization; (iii) analyze *VmHAD* gene expression during Vm1 infection of apple branches. The results of this study will enhance the understanding of the biological characteristics and functional roles of HAD enzymes in pathogenic fungi and may contribute to the development of effective strategies for apple Valsa canker management.

## 2. Materials and Methods

### 2.1. Fungal Preparation

The pathogen of Vm1 that causes apple Valsa canker was provided by the Laboratory of Plant Virology and Molecular Biology, College of Plant Protection, Gansu Agricultural University. The strain of Vm1 was cultured on potato dextrose agar (PDA) at 25 °C for 5 days for further experiments.

### 2.2. Fungal Total RNA Extraction and First-Strand cDNA Synthesis

For the total RNA extraction, the fresh mycelia of Vm1 were collected 5 days after inoculation on PDA media, and frozen with liquid nitrogen and stored at −80 °C for further experiments. The specific method for the total RNA extraction was used according to the manufacturer’s instructions for the Fungal RNA Extraction kit (OMEGA Bio-Tek, Norcross, GA, USA). The total RNA purity was assessed by OD260/280 and OD260/230 ratios. Integrity was checked by agarose gel electrophoresis. Thereafter, high-quality RNA samples were used for cDNA synthesis in accordance with the manufacturer’s instructions for the First-Strand cDNA Synthesis kit (Thermo Fisher Scientific, Waltham, MA, USA).

### 2.3. Haloacid Dehalogenase Gene (VmHAD) of Valsa mali Vm1 Cloning and Characterization

For cloning the full-length cDNA sequence of the *VmHAD* gene from Vm1, a fragment of the *VmHAD* gene was obtained from the transcriptome data in our previous work and used to design the primers (*VmHAD-CF-F* and *VmHAD-CF-R*). The core fragment of the *VmHAD* gene was cloned by using the cDNA of Vm1 as the template, and the *VmHAD-CF-F* and *VmHAD-CF-R* as primers to amplify. Thereafter, the synthesis of 5′ RACE and 3′ RACE cDNA fragments was carried out using the SMARTer^®^ RACE 5′/3′ Kit (Takara Bio. Inc., Dalian, China), and the primers of *VmHAD-5-1* and *VmHAD-5-2*, and *VmHAD-3-1* and *VmHAD-3-2* were designed to clone the 5′ RACE and 3′ RACE cDNA fragments, respectively. The full-length cDNA sequence of the *VmHAD* gene was verified using the primers of *VmHAD-FL-F* and *VmHAD-FL-R*. Primers were designed using Primer Premier 5.0 software, and synthesized and sequenced by Tsingke Biotechnology Co., Ltd., Beijing, China (Table S1). The open reading frame (ORF) of the *VmHAD* gene was analyzed by ORF Finder (https://www.ncbi.nlm.nih.gov/orffinder/) (accessed on 15 May 2025) and then the corresponding amino acid sequences were deduced by DNAMAN 8.0 (Lynnon Biosoft, San Ramon, CA, USA). For the further analysis and characterization of the VmHAD protein, the multiple sequences of HAD amino acids from 7 species of plant pathogens were aligned and performed using DNAMAN 8.0. A maximum likelihood (ML) phylogenetic tree was constructed using the Mega 7.0 with a bootstrap value of 1000 replicates, according to the VmHAD and other reference HAD amino acid sequences.

### 2.4. VmHAD Bioinformatic Analysis

The related genes sequence was searched using BLAST in the NCBI databank (http://www.ncbi.nlm.nih.gov/sutils/genom_table.cgi) (accessed on 15 May 2025); the physicochemical properties of the VmHAD protein were analyzed using EXPASY (https://web.expasy.org/protparam/) (accessed on 20 August 2025); the conservative structure domain was predicted using the NCBI database (https://www.ncbi.nlm.nih.gov/Structure/cdd/wrpsb.cgi) (accessed on 15 May 2025); the membrane structure domain, signal peptide, phosphorylation sites, and protein secondary structure were analyzed using the TMHMM Server v. 2.0 (https://services.healthtech.dtu.dk/services/TMHMM-2.0/) (accessed on 15 May 2025), SignalP-5.0 Server (https://services.healthtech.dtu.dk/services/SignalP-5.0/) (accessed on 15 May 2025), NetPhos 3.1 Server (https://services.healthtech.dtu.dk/services/NetPhos/) (accessed on 15 May 2025), and SOPMA (https://npsa.lyon.inserm.fr/cgibin/npsa_automat.pl?page=/NPSA/npsa_sopma.html) (accessed on 20 August 2025), respectively; and the tertiary structure was constructed using the SWISS-MODEL (https://swissmodel.expasy.org/) (accessed on 15 May 2025).

### 2.5. VmHAD Gene Recombinant Vector Construction and Transformation

To monitor the subcellular localization of the VmHAD protein, the primers (*VmHAD-C-F* and *VmHAD-C-R*) were designed according to the full-length cDNA sequence of the *VmHAD* gene and used to clone the coding region (CDS) of the *VmHAD* gene (*VmHAD-CDS*). The primers (*EF-VmHAD-F* and *EF-VmHAD-R*) were designed to amplify the expression fragment of *EF-VmHAD*. Thereafter, *EF-VmHAD* was ligated into a *pBWA(V)HS-GLosgfp* plasmid using *BsaI/Eco31I* sites, and finally the recombinant plasmid (*pBWA(V)HS-EF-VmHAD-GLosgfp*) was transformed into *E. coli* DH5α. Finally, the positive single clones were selected for PCR verification using the primers of *V-VmHAD-HS* and *V-VmHAD-D1*. The verified positive single clones were used to extract the plasmids and then transformed into *Agrobacterium tumefaciens* GV3101. Thereafter, the single clones were observed and picked for PCR detection. Therefore, the cultured liquid of the verified clones was prepared for *A. tumefaciens* GV3101 transformation. Primers were designed using Primer Premier 5.0 software, and synthesized and sequenced by Tsingke Biotechnology Co., Ltd., Beijing, China (Table S1).

### 2.6. VmHAD Subcellular Location Analysis by Transient Expression 

The transient expression of the VmHAD protein was performed in *Nicotiana benthamiana*. The *A. tumefaciens* GV3101 solutions carrying *pBWA(V)HS-EF-VmHAD-GLosgfp* and *pBWA(V)HS-GLosgfp* were injected into the leaves of 4-week-old *N. benthamiana* seedlings with three replications. Thereafter, the transformed *N. benthamiana* seedlings were incubated at 25 °C for 3 days after injection, and then fluorescence was observed with a confocal microscope (Nikon C2-ER, Tokyo, Japan).

### 2.7. VmHAD Gene Expression Characteristics Analysis During the Valsa mali Vm1 Infection Process by RT-qPCR

Healthy and uniform detached apple branches (cultivars, ‘Fuji’) with a diameter of approximately 6–7 mm were prepared for inoculation with Vm1 and *VmHAD* gene expression analysis during the infection process by RT-qPCR. The apple branches were disinfected with 1% sodium hypochlorite solution (NaOCl), rinsed with sterile water three times, and finally air-dried for further experiments. The cut edges of apple branches were sealed with paraffin wax and wounds were made on the surface. Thereafter, the disks of Vm1 were inoculated on the wounds of the detached apple branches, whereas the PDA disks without inoculation were used as controls. The inoculated branches were kept at 25 °C for 7 days. The tissue around the lesions of the branches in the treatment and control groups were collected after 1, 3, 5, and 7 days with a 2 day interval after inoculation, and stored at −80 °C for subsequent experiments. The total RNA extraction and First-Strand cDNA synthesis were performed according to the manufacturer’s instructions for the E.Z.N.A.^®^ Plant RNA Kit (Tiangen Biotechnology, Beijing, China) and the First-Strand cDNA Synthesis kit (Thermo Fisher Scientific, Waltham, MA, USA), respectively. The cDNA from the treatment and control groups was used as the template for determining the *VmHAD* gene expression characteristics after inoculation during the Vm1 infection process using the TB Green^®^ Premix Ex Taq^™^ II kit (Takara, Dalian, China). Glucose–hexaphosphate dehydrogenase (*G6PDH*) (KC248180) and cytochrome enzyme (*CYP*) (KC248178) were selected as the internal reference genes. Primers for RT-qPCR determination were designed using Primer Premier 5.0 and named as *q-VmHAD-F* and *q-VmHAD-R*, *G6PDH-F* and *G6PDH-R*, *CYP-F* and *CYP-R*, and synthesized by Tsingke Biotechnology Co., Ltd., Beijing, China (Table S1). The stability of *G6PDH* and *CYP* was validated, and their melt curve analysis was evaluated before using them for *VmHAD* gene expression level determination. The samples in each treatment and the control were collected from three independently inoculated (treatment) or un-inoculated (control) branches, and each sample had three replications.

### 2.8. Statistical Analysis

The data for the *VmHAD* gene expression characteristics in the present study were calculated using the 2^−ΔΔCt^ method and analyzed using one-way ANOVA using SPSS 20.0 (SPSS Inc., Chicago, IL, USA). The Kolmogorov–Smirnov method and *p*-value were used to check the normality of the sample distribution. The significant differences among the treatments were determined using the Duncan’s multiple range test at *p* < 0.05.

## 3. Results

### 3.1. Full-Length cDNA Sequence of VmHAD Gene Cloning and Characterization

**Figure 1:**
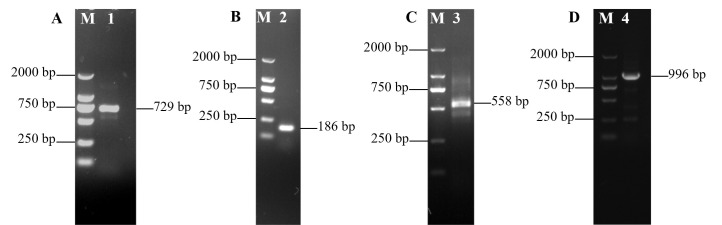
Full-length cDNA sequence of *VmHAD* gene of *V. mali* Vm1 cloning and characterization: M: D2000 marker; (**A**): lane 1, core fragment; (**B**): lane 2, 5′ RACE fragment; (**C**): lane 3, 3′ RACE fragment; (**D**): lane 4, full-length cDNA fragment verified.

**Figure 2:**
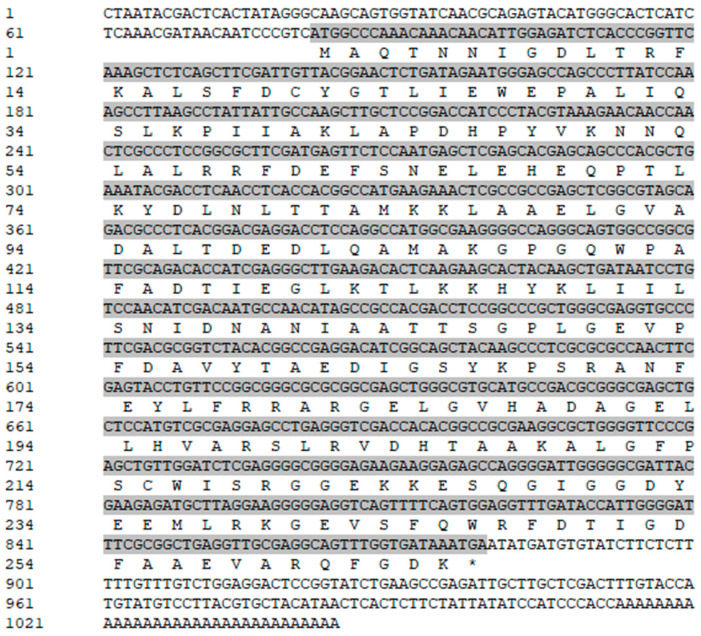
Full-length cDNA and amino acid sequences of *VmHAD* gene of *V. mali* Vm1. Gray shaded sequences represent the ORF region; “*” represents the stop codon.

**Figure 3:**
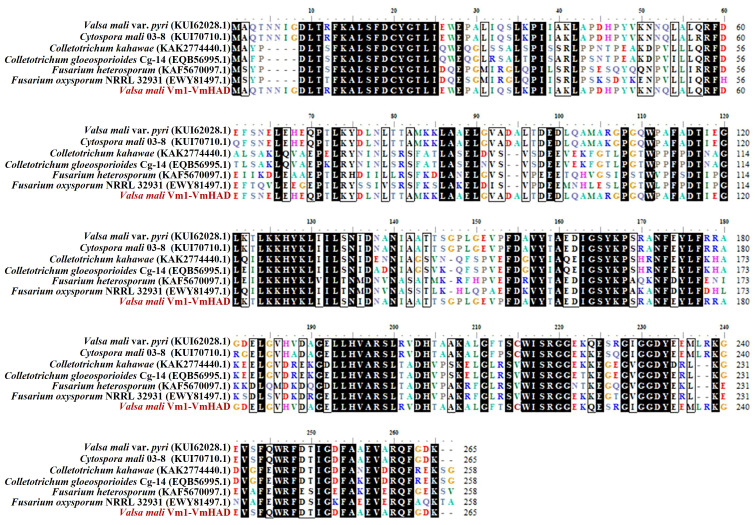
Amino acid sequence alignment of VmHAD protein. Multiple sequences were aligned using DNAMAN 8.0.

**Figure 4:**
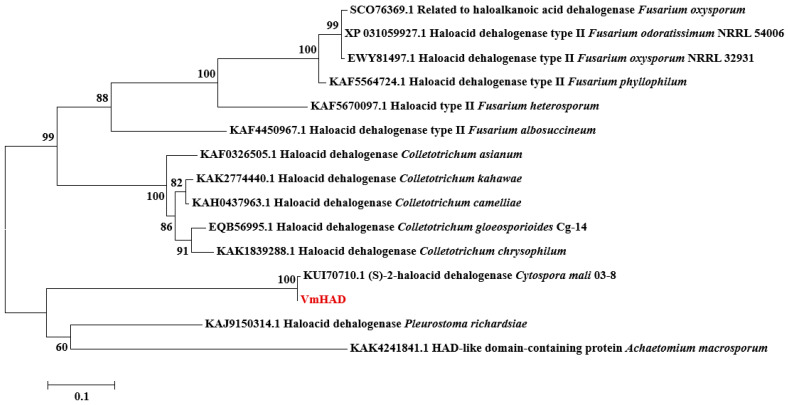
Phylogenetic tree construction of VmHAD protein. A maximum likelihood (ML) phylogenetic tree was constructed with a bootstrap value of 1000 replicates.

The core fragment of the *VmHAD* gene from Vm1 was amplified by RT-PCR, with a length of 729 bp after it was sequenced (Figure 1A). Meanwhile, a 186 bp 5′ fragment (Figure 1B) and a 558 bp 3′ fragment (Figure 1C) were successfully cloned using the SMARTer^®^ RACE 5′/3′ Kit and verified by sequencing. The full-length cDNA sequence of the *VmHAD* gene was ultimately obtained by splicing the 5′ and 3′ end sequences of the *VmHAD* gene with the fragment, producing a length of 1044 bp, which contained a complete ORF of 798 bp that encoded 265 amino acids (Figure 2). Thereafter, a 996 bp fragment was amplified to verify that the full-length cDNA sequence of the *VmHAD* gene was obtained correctly (Figure 1D). In addition, the VmHAD protein exhibited the highest similarity to *Cytospora mali* 03-8 (Figure 3) based on complete amino acid sequence analysis. The phylogenetic tree analysis revealed that it was closest to the (S)-2-haloacid dehalogenase (accession number KUI70710.1) of *C. mali* 03-8, with clustering in the same clade with a bootstrap support rate of 100% (Figure 4).

### 3.2. VmHAD Bioinformation Analysis

**Figure 5:**
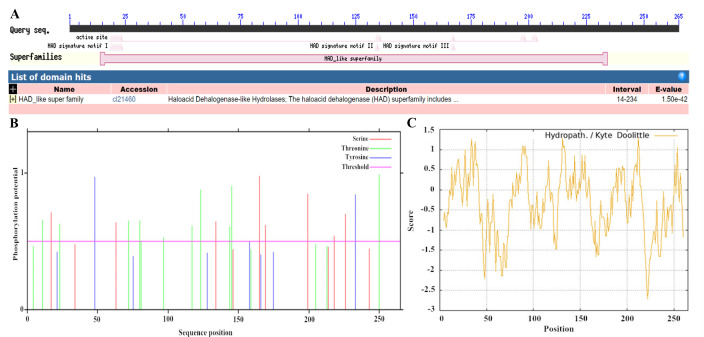
Bioinformation of VmHAD protein. (**A**): Conserved domain analysis; (**B**): phosphorylation sites prediction; (**C**): hydrophilic and hydrophobic properties analysis.

**Figure 6:**
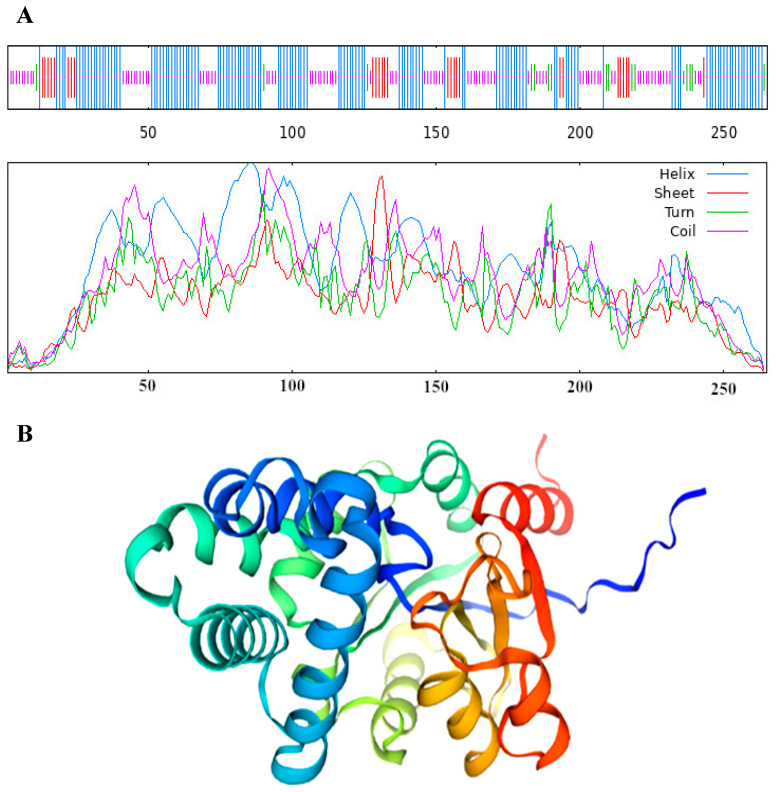
Secondary structure (**A**) and tertiary structure (**B**) of VmHAD prediction.

The results revealed that the VmHAD protein comprises 265 amino acids, with a molecular formula of C_1317_H_2049_N_357_O_395_S_6_ and an approximate molecular weight of 29.4 kDa. The theoretical isoelectric point (pI), aliphatic index (AI), grand average of hydrophilicity (GRAVY), and instability index were 5.47, 83.36, −0.358, and 38.20, respectively. The predominant amino acids included alanine (11.7%), leucine (10.6%), and glycine (7.9%), and there were also 38 negatively charged residues (Asp + Glu) and 31 positively charged residues (Arg + Lys) (Table 1). The conserved domain structure analysis revealed that the VmHAD protein had a particularly matched HAD-like junction domain at positions 14-234 of the amino acid sequences and belongs to the L-2-haloacid dehalogenase family within the HAD-like superfamily (Figure 5A). However, the VmHAD protein did not contain a signal peptide and transmembrane regions. Furthermore, the phosphorylation site prediction revealed that the VmHAD protein contains 34 potential phosphorylation sites, including 12 serine (Ser, S) phosphorylation sites at positions S17, S34, S63, S134, S146, S165, S169, S199, S214, S218, S226, and S243; 14 threonine (Thr, T) phosphorylation sites at positions T4, T11, T23, T72, T80, T81, T97, T117, T123, T144, T145, T159, T205, and T250; and 8 tyrosine (Tyr, Y) phosphorylation sites at positions Y21, Y48, Y75, Y128, Y158, Y166, Y175, and Y233 (Figure 5B). The hydrophilicity analysis also confirmed that it is a hydrophilic protein (Figure 5C). The predictive secondary structure of the VmHAD protein indicated that there are four spatial conformations. Among them, the α-helix has the highest proportion, accounting for 49.06% of the amino acid residues, followed by random coils and extended chains accounting for 34.72% and 10.57%, respectively, whereas the β-sheets had the lowest proportion at 5.66% (Figure 6A). The tertiary structure of the VmHAD protein indicated that it encompasses amino acids from the 1st to the 265th position. The similarity between the sequence and the template sequence was 96.98%, with a coverage rate of 0.93 (Figure 6B).

### 3.3. Subcellular Localization Vector of VmHAD Gene Construction and Transformation

**Figure 7:**
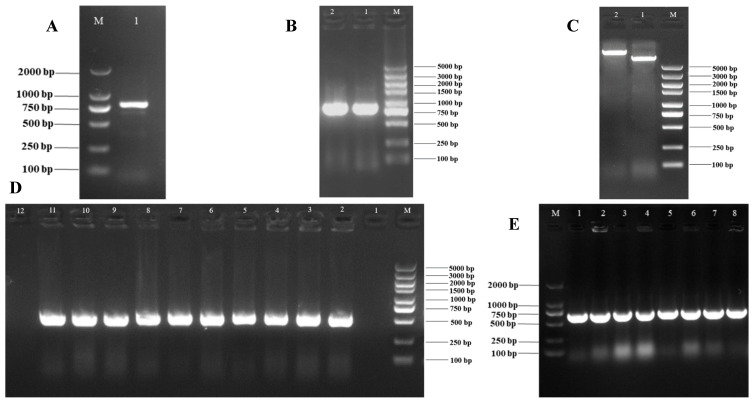
Subcellular localization vector of *VmHAD* gene construction and transformation. M: D2000 and D5000 markers; (**A**): CDS fragment of *VmHAD* gene cloning, where line 1 represents CDS fragment; (**B**): expression fragment cloning, where lines 1–2 represent the expression fragment of *EF-VmHAD*; (**C**): recombinant plasmid construction, where line 1 represents the empty plasmid of *pBWA(V)HS-GLosgfp* and line 2 represents the recombinant plasmid of *pBWA(V)HS-EF-VmHAD-GLosgfp*; (**D**): recombinant plasmid transformation, where line 1 represents the transformed empty plasmid (*pBWA(V)HS-GLosgfp*) of *E. coli* DH5α clone detected by PCR verification, lines 2–11 represent the transformed recombinant plasmid (*pBWA(V)HS-EF*-*VmHAD-GLosgfp*) of *E. coli* DH5α clones, and line 12 represents the blank control; (**E**): *A. tumefaciens* GV3101 clone detection, where lines 1–8 represent the transformed recombinant plasmid (*pBWA(V)HS-EF-VmHAD-GLosgfp*) of *A. tumefaciens* GV3101 clones detected by PCR verification.

For monitoring the subcellular localization of the *VmHAD* gene, a 798 bp fragment of CDS was obtained and sequenced (Figure 7A). Thereafter, an 835 bp expression fragment (*EF-VmHAD*) was amplified (Figure 7B) and ligated into the double-digested *pBWA(V)HS-GLosgfp* plasmid (Figure 7C). Finally, a single band with a length of approximately 680 bp was obtained in each of transformed clones after successful transformation into *E. coli* DH5α (Figure 7D, lanes 2–11), whereas the clone that transformed the empty plasmid did not yield any bands (Figure 7D, lane 1), and also the blank control showed no bands (Figure 7D, lane 12). Finally, a 680 bp fragment was amplified from each of the eight well-growing single clones after transformation into *A. tumefaciens* GV3101 (Figure 7E, lanes 1–8).

### 3.4. VmHAD Subcellular Location Analysis

**Figure 8:**
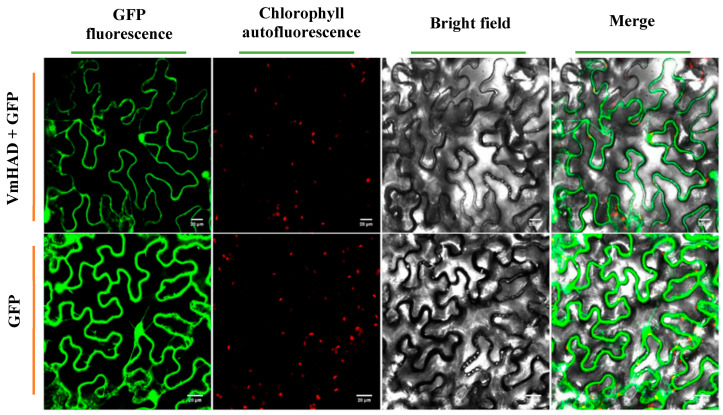
Localization of VmHAD protein expressed transiently in *N. benthamiana* leaves at the subcellular level. The size of the scale bars represents 20 µm.

The expression location of the VmHAD protein in *N. benthamiana* leaves was observed at the subcellular level using laser confocal microscopy. The results showed that the VmHAD protein was possibly located in both the nucleus and cytoplasm after being injected into the *N. benthamiana* leaves using the transient expression system (Figure 8).

### 3.5. VmHAD Gene Expression Characteristics Analysis 

**Figure 9:**
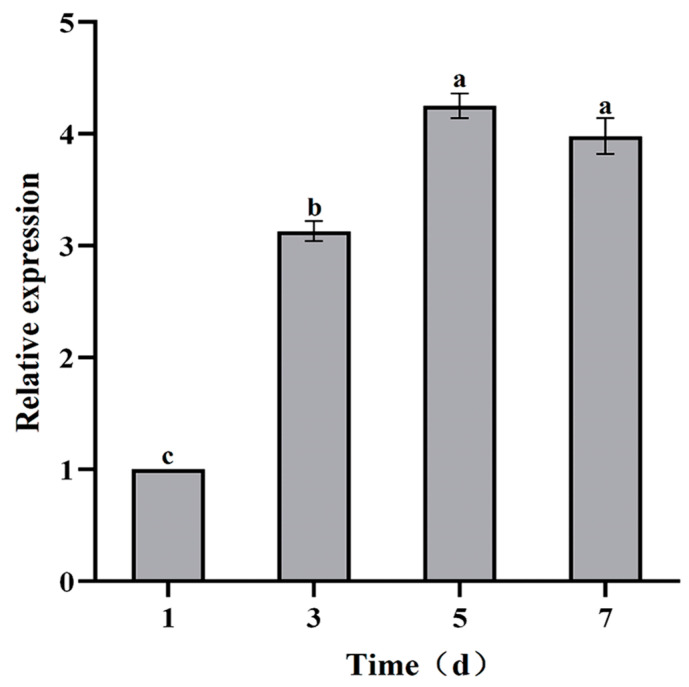
The expression characteristics of *VmHAD* gene analysis at different time points during the *V. mali* Vm1 infection process. Line bars represent the standard errors (SEs) of the means of replications, and the different letters indicate the significant difference among the different time points during the infection process at *p <* 0.05 level according to Duncan’s new multiple range test.

Compared with the control, the *VmHAD* gene was significantly expressed during the infection process of Vm1 on detached apple branches at different time points, whereas an expression level was not found in the control branches (no inoculation). The expression level of the *VmHAD* gene was significantly increased during Vm1 infection, with significant difference from day 1 to 5 after inoculation with Vm1 on the detached apple branches, and stability expressed at days 5 and 7. The relative expression level of the *VmHAD* gene was increased by 3.13-, 4.25-, and 3.98-fold at 3, 5, and 7 days after inoculation in comparison to day 1, with the highest expression level after 5 days (Figure 9).

## 4. Discussion

HAD-like hydrolases have been found in organisms with 479,051 sequences in databases (InterPro IPR023214) and 33 major families [45,46]. The number of HAD genes that has been reported in different bacteria has ranged from 10 to 20, as well as 100 in humans, 115 in *Arabidopsis thaliana* (InterPro database), and more than 45 in *Saccharomyces cerevisiae* in their individual genomes [47]. However, the majority of the HADs’s biological and structural characteristics and even their functions remain uncharacterized [48]. In our present study, a novel *VmHAD* gene from Vm1 with full-length cDNA sequences of 1044 bp were cloned using RACE technology, and its genetic relationship was closest to the (S)-2-halogenated dehalogenase (accession number KUI70710.1) of *C*. *mali* 03-8, with a bootstrap support rate of 100%, which belongs to the L-2-haloacid dehalogenase family of the HAD hydrolase superfamily. Previous studies revealed that 2-haloacid dehalogenase in microorganisms is widespread in nature, and has been classified into four types including the D-2-haloacid dehalogenase, L-2-haloacid dehalogenase, configuration-inverting DL-2-haloacid dehalogenase, and configuration-retaining DL-2-haloacid dehalogenase, according to the substrate specificities and product configurations [49]. To date, the biochemical characterization of 2-haloacid dehalogenases has been studied, including the L-2-haloacid dehalogenase from the thermophilic archaeon *Sulfolobus tokodaii* [50], the DL-2-haloacid dehalogenase gene from *Burkholderia cepacia* [51], and even a novel L-2-haloacid dehalogenase (L-2-DhlB) (25 kDa) has been isolated from the strain of *Ancylobacter aquaticus* UV5, and it was found that it belongs to the family of L-2-haloacid dehalogenase [52]. However, specific functions, such as their role in pathogenicity, have not been fully explored recently.

In addition, our study found that the ORF of the *VmHAD* gene was 798 bp, corresponding to 265 amino acids, and with a particularly matched junction domain that was HAD-like. Kumar et al. (2016) [52] investigated the L-2-DhlB of *A. aquaticus* UV5 with a putative conserved domain of a hypothetical HAD-like superfamily and subfamily IA, and a 693 bp ORF sequence corresponding to 230 amino acids; Ren et al. (2023) found that the HAD-like superfamily *Nem1* gene from the pathogen of *B. dothidea* contains a transmembrane domain and a characteristic HAD-like domain [44]. Additionally, the cDNA fragment of *MoNEM1* from *Magnaporthe oryzae* that contains an ORF encoding 537 amino acids and a conserved HAD-like superfamily domain was also investigated [53]. However, the differences in ORF length and domain structure in our present study and previous studies may indicate the differences in their potential function in different organisms, especially the pathogenic functions.

Phosphorylation is an important post-translational modification process that plays a significant role in regulating the activity, stability, and subcellular localization of proteins. In our present study, we predicted that the phosphorylation site of the VmHAD protein contains 34 potential phosphorylation sites, and found that it is possibly located in both the nucleus and cytoplasm of *N. benthamiana* at the subcellular level. Consistently, Lee et al. (2022) reported that the haloacid dehalogenase-like phosphatase AtHAD1 was involved in repressing the ABA response and expressed in the *Arabidopsis* nucleus and cytoplasm [54]. Thus, we hypothesis that this dual localization indicates that the VmHAD protein could participate in various metabolic processes, such as the possible functions of the *VmHAD* gene involved in modulating nuclear signaling pathways or in cytoplasmic metabolic processes. However, work related to the quantitative assessment and the localization of nuclear and cytoplasmic markers will be performed in the future.

Furthermore, we found that the *VmHAD* gene was significantly expressed during the Vm1 infection process on the detached apple branches. The expression level was significantly increased with infection process time increased from day 1 to 5, and with significant differences from 3 to 7 days in comparison to day 1. Thus, our result indicates that the function of the *VmHAD* gene may play a potential role in the pathogenicity of Vm1. Ren et al. (2023) found that the *Nem1* homolog in *B. dothidea* was significantly upregulated during the infection process, which indicates that the *Nem1* gene is important for the pathogenicity of *B. dothidea* [44]. Other previous studies found that *Nem1* was involved in regulating mycelial growth and conidiation but not in conidial morphology or germination, including the filamentous fungi of *Aspergillus fumigatus*, *M*. *oryzae*, and *F. graminearum* [53,55,56,57]. Zhang et al. (2023) found that the expression level of *FoHAD-type II* plays an important role in the pathogenic process of *F. oxysporum* f. sp. *momordicae* [42]. Fang et al. (2014) found that the *FOXG-07877* gene that codes for the HAD superfamily of hydrolases showed a downregulated expression in the strawberry wilt pathogen (with a lower-toxin strain of *F. oxysporum* f. sp. *fragariae*) by analyzing the different expression genes in different pathogenic strains at the protein level in comparison to the higher toxin strain [43]. In addition, Garcia-Martinez et al. (2014) reported that the *cutA* gene from *F. fujikuroi* that encodes the protein of the haloacid dehalogenase family and its functions were involved in osmotic stress and glycerol metabolism [41]. However, the specific pathogenic mechanisms and pathways for *HAD* genes in plant pathogens are little researched; in particular, the molecular pathogenic mechanisms and regulatory pathways involving VmHAD virulence in Vm1 are unknown and will be further studied in future.

## 5. Conclusions

In conclusion, a detailed characterization of a novel haloacid dehalogenase superfamily gene (*VmHAD*) from Vm1 was completed and monitored at the molecular and subcellular levels. Our results found that the VmHAD protein was closest to the (S)-2-halogenated dehalogenase of *C. mali* 03-8, which belongs to the L-2-haloacid dehalogenase of the HAD hydrolase superfamily. In addition, it was possibly located in both the nucleus and cytoplasm after being transformed into the *N. benthamiana* at the subcellular level. We also found that VmHAD expression was induced during Vm1 infection. Our results indicate that the function of the VmHAD protein was related to the Vm1 infection process and may play a potential role in the pathogenicity of Vm1. However, the specific pathogenic functions and mechanisms for the VmHAD protein will be further identified using gene knocking out technology and the quantitative assessment of its localization will be performed in future work.

## Figures and Tables

**Table 1:** Physicochemical properties of VmHAD protein.

Physicochemical Properties	VmHAD Protein
Number of amino acids	265
Relative molecular mass	29.4 kDa
Molecular formula	C_1317_H_2049_N_357_O_395_S_6_
Theoretical isoelectric point (pI)	5.47
Total number of positively charged residues (Arg + Lys)	31
Total number of negatively charged residues (Asp + Glu)	38
Grand average of hydropathicity (GRAVY)	−0.358
Aliphatic index (AI)	83.36
Instability index (II) (<40 stable, >40 unstable)	38.20

## Data Availability

The original contributions presented in this study are included in the article, and further inquiries can be directed to the corresponding author.

## Supplementary Materials

The following supporting information can be downloaded at: https://www.mdpi.com/article/10.3390/jof11120827/s1, Table S1. Primers for *VmHAD* gene cloning, subcellular localization, and expression characteristics determination.
