# Supplementary material for: Identification, Subcellular Localization, and Infection-Related Expression of a Novel Haloacid Dehalogenase Gene (VmHAD) from Valsa mali Vm1
Source: J Fungi (Basel). 2025 Nov 23;11(12):827. doi: 10.3390/jof11120827 (PMC12733955; doi:10.3390/jof11120827)
Supplement: Supplementary file 1 [file jof-11-00827-s001.zip › jof-3870737-supplementary.pdf]

Table S1. Primers for *VmHAD* gene cloning, subcellular localization and expression characteristics determination

Fragments	Primers Name	Primers Sequence
VmHAD-CF	*VmHAD-CF-F*	TGTTACGGAACTCTAATAGAATGGG
	VmHAD-CF-R	AAACTGCCTCGCAACCTCA
VmHAD-5′ RACE	*VmHAD-5-1*	GCTCATTGGAGAACTCGTC
	VmHAD-5-2	GGCTGGCTCCCATTCTATT
VmHAD-3′ RACE	*VmHAD-3-1*	ACGCTCAAGAAGCACTAC
	VmHAD-3-2	CAAGCTGATAATCCTGTCC
VmHAD-FL	*VmHAD-FL-F*	AAGCAGTGGTATCAACGCAGAG
	VmHAD-FL-R	TGAGTTATGTAGCACGTAAGGA
VmHAD-CDS	*VmHAD-C-F*	ATGGCCCAAACAAACAACATTGGAG
	VmHAD-C-R	TCATTTATCACCAAACTGCCTCGCA
EF-VmHAD	*EF-VmHAD-F*	AACACGGGGGACTTTGCAACatggcccaaacaaaca acattggagatctc
	EF-VmHAD-R	CCTGAAGCGGCCGCTGTACAtttatcaccaaactgcct cgcaacctc
V-VmHAD	*V-VmHAD-HS*	TTCATTTGGAGAGAACACGGGGGAC
	V-VmHAD-D1	CAACAGCTCGGGAACCCCAG
q-VmHAD	*q-VmHAD-F*	TCCAATGAGCTCGAGCACGA
	q-VmHAD-R	CAATCTCGGCTTCAGATACCG
G6PDH	*G6PDH-F*	TCAGAACAAGTTCGAGGGCGACAA
	G6PDH-R	TGAGGGCAATAGAGGGCTTGTTCA
CYP	*CYP-F*	GCAAGTCCATCTTCGGTGAG
	CYP-R	TGGGAGCCGTTGGTGTT
